# EVIDENCE OF AGE BIAS ON THE ALTERNATIVE MODEL OF PERSONALITY DISORDERS

**DOI:** 10.1093/geroni/igad104.3019

**Published:** 2023-12-21

**Authors:** Lisa Stone-Bury, Alyssa Premovich, Daniel Segal

**Affiliations:** University of Colorado at Colorado Springs, Colorado Springs, Colorado, United States; University of Colorado at Colorado Springs, Colorado Springs, Colorado, United States; University of Colorado at Colorado Springs, Colorado Springs, Colorado, United States

## Abstract

**Introduction:**

Previous research established substantial age bias on personality disorder (PD) diagnostic criteria for older adults. The Alternative Model of Personality Disorders (AMPD) is a new model of PDs proposed in DSM-5’s Section III that measures PDs dimensionally along two criteria: personality functioning with four domains (measured by the Levels of Personality Functioning Scale; LPFS) and pathological personality traits with five domains (measured by the Personality Inventory for DSM-5; PID-5). This study examined age bias on the AMPD in a cross-sectional design. Method: Older (n = 200) and younger adults (n = 213) completed the LPFS-Self-Report (LPFS-SR) and the PID-5-Brief Form (PID-5-BF).

**Results:**

Differential Item Functioning (DIF) analyses were computed on items of the LPFS-SR and PID-5-BF. An item exhibits DIF if two age groups with similar levels of an underlying scale have different probabilities of endorsing an item. Overall, 18 of 80 items on the LPFS-SR demonstrated large DIF. The Empathy (50% of items) and Intimacy (25% of items) domains were most aged biased. For the PID-5-BF, 10 of 25 items showed large DIF. All five domains demonstrated large age bias, with Psychoticism (100% of items) most impacted. Across both DIF models, items showed evidence of being biased for (disproportionately high endorsement of an item) or against (disproportionately low endorsement) older adults.

**Discussion:**

Findings indicate meaningful age bias on both AMPD diagnostic criteria, indicating that older adults are at-risk of being under- and over-diagnosed with PDs under the AMPD. Revision of the AMPD is warranted to achieve age neutrality.

